# Characteristics, management and attainment of lipid target levels in diabetic and cardiac patients enrolled in Disease Management Program versus those in routine care: LUTZ registry

**DOI:** 10.1186/1471-2458-9-280

**Published:** 2009-08-04

**Authors:** Kurt Bestehorn, Christina Jannowitz, Barbara Karmann, David Pittrow, Wilhelm Kirch

**Affiliations:** 1 Medical Department, MSD Sharp & Dohme GmbH, Haar, Germany; 2 Institute for Clinical Pharmacology, Medical Faculty, Technical University, Dresden, Germany; 3 Medical Department, Essex Pharma GmbH, Munich, Germany

## Abstract

**Background:**

Since 2002 the sick funds in Germany have widely implemented disease management programs (DMPs) for patients with type 2 diabetes mellitus (DM) and coronary heart disease (CHD). Little is known about the characteristics, treatment and target attainment lipid levels of these patients enrolled in DMPs compared to patients in routine care (non-DMP).

**Methods:**

In an open, non-interventional registry (LUTZ) in Germany, 6551 physicians documented 15,211 patients with DM (10,110 in DMP, 5101 in routine care) and 14,222 (6259 in DMP, 7963 in routine care) over a follow-up period of 4 months. They received the NCEP ATP III guidelines as a reminder on lipid level targets.

**Results:**

While demographic characteristics of DMP patients were similar to routine care patients, the former had higher rates of almost all cardiovascular comorbidities. Patients in DMPs received pharmacological treatment (in almost all drug classes) more often than non-DMP patients (e.g. antiplatelets: in DM 27.0% vs 23.8%; in CHD 63.0% vs. 53.6%). The same applied for educational measures (on life style changes and diet etc.). The rate of target level attainment for low density lipoprotein cholesterol (LDL-C) < 100 mg/dl was somewhat higher in DMP patients at inclusion compared to non-DMP patients (DM: 23.9% vs. 21.3%; CHD: 30.6% vs. 23.8%) and increased after 4 months (DM: 38.3% vs. 36.9%; CHD: 49.8% vs. 43.3%). Individual LDL-C target level attainment rates as assessed by the treating physicians were higher (at 4 months in DM: 59.6% vs. 56.5%; CHD: 49.8% vs 43.3%). Mean blood pressure (BP) and HbA_1c _values were slightly lowered during follow-up, without substantial differences between DMP and non-DMP patients.

**Conclusion:**

Patients with DM, and (to a greater extent) with CHD in DMPs compared to non-DMP patients in routine care have a higher burden of comorbidities, but also receive more intensive pharmacological treatment and educational measures. The present data support that the substantial additional efforts in DMPs aimed at improving outcomes resulted in quality gains for achieving target LDL-C levels, but not for BP or HbA_1c_. Longer-term follow-up is needed to substantiate these results.

## Background

Disease management typically refers to multidisciplinary efforts to improve the quality and cost-effectiveness of care for selected patients suffering from chronic conditions [1]. An explicit systematic population-based approach is applied to identify persons at risk, to intervene with specific programs of care (disease management programs, DMP), and to measure clinical and other outcomes [2]. These programs, however, are widely heterogeneous across health-care systems, and difficult to compare across interventions [3]. In the German statutory health insurance in 2002 some of the worlds largest DMPs ‐ without a pilot evaluation phase ‐ were launched, initially for type 2 diabetes mellitus (DM), breast cancer and coronary heart disease (CHD), subsequently also for type 1 DM and asthma/COPD [4,5]. The nationwide DMPs have been implemented through sick funds, which cover around 88% of the general population, and to date, 14,000 of such programs have been accredited [6]. Physicians that enrol voluntarily in such programs are legally obliged to follow certain evidence-based clinical practice guidelines and to document individual patients comprehensively. As an incentive, sick funds receive a higher remuneration for DMP patients from the risk structure compensation pool and the patient can expect to be provided with higher-quality and more cost-effective care [4].

While sick funds are obliged by law to intermittently carry out DMP evaluations, such procedures are performed without a control group, are strictly limited to the accreditation period and to a relative lean core data set [7]. Criteria for evaluation include medical issues, economic issues and quality of life. Until now, not much is known about data quality or outcomes [8]. While according to the German Ministry of Health analyses up to 2005 generally indicate good patient management [9], the Federal Physician Association (Kassenärztliche Bundesvereinigung) stated that there is a substantial need for additional funding for guideline-oriented therapy [10].

DMPs for DM and/or CHD consider lipid lowering therapy to be an integral part of the treatment [7]. Low-density lipoprotein cholesterol (LDL-C) is acknowledged as a pivotal parameter for assessment of the success of lipid-lowering therapy, and patients with DM or CHD have a common target goal of < 100 mg/dl [11]. Therefore, this LDL-C threshold can be used for a joint evaluation for both patient groups. Further, target level attainment rates of blood pressure or HbA_1c _targets lend themselves for outcomes research.

The present registry in the primary care setting aimed to address the following questions: (1) Do patients in DMPs, separated by indication (DM and CHD) differ from patients not treated in DMPs (routine care) in terms of demographic characteristics, comorbidities/risk factors, or treatment? (2) Can during a follow-up period of 4 months, by participation in the registry and dissemination of guidelines, treatment be quantitatively and qualitatively improved? (3) Are LDL-C, blood pressure and glycosylated haemoglobin A_1c _(HbA_1c_) target level attainment rates higher in patients within DMPs compared to patients in routine care (non-DMP)? (4) Do target level attainment rates differ between the DMPs for DM and CHD?

## Methods

### Study design and patients

The present study (Lipidmanagement und Therapieziel-Erreichung bei Patienten mit KHK und/oder Diabetes mellitus, LUTZ) was designed as a prospective observational, non-interventional, multicentre registry at 6551 sites throughout Germany, and performed between March 2006 and April 2007. Practice-based family physicians and internists (serving as general physicians, or having a speciality in diabetology or cardiology) were invited to participate, irrespective of whether they took part in DMPs or not.

In addition to the case report forms (CRFs), physicians were informed about authoritative guidelines concerning LDL-C target levels for patients with CHD and/or DM, respectively. They received a printed summery of the updated National Cholesterol Education Panel Adult Panel III (NCEP ATP III) guidelines [11].

They were requested to include 6 male or female outpatients with CHD and/or DM. In practices that participated in a DMP, this sample was to be balanced (3 patients in any DMP, 3 non-DMP patients). Further, physicians had to ensure that after study initiation the next 6 eligible patients had to be documented sequentially to avoid selection bias. Patients had to have hypercholesterolaemia as diagnosed by the treating physician, a history of CHD and/or DM, and should be on chronic treatment with a statin at inclusion. No other inclusion or exclusion criteria applied.

The study was approved by the certified ethics committee of the Bavarian Physicians Chamber. Patient data protection was fully ensured. As part of the quality assurance process, an on-site audit was performed in 50 centres. For the complete documentation of each patient, physicians received a small remuneration of 20 € per patient, which is standard for this type of study.

### Physician characteristics

In connection with the agreement form, physicians were requested to report their year of birth, gender, and start date of practice-based service. Further, they noted whether they practised in a rural area, a small or large town, and the number of patients per quarter, the number of physician colleagues in their practice, the participation of their practice in one or more of the DMPs, and the number of patients in the DMPs for CHD or DM (if applicable).

### Assessments

Two visits were foreseen (baseline and 4-month follow up). At entry, physicians recorded patient characteristics (weight, height), demographic data (year of birth, gender), inclusion in the DMPs for CHD or DM (if applicable), and the inclusion diagnosis (CHD, DM). Further, they documented cardiac risk factors (CHD with details on type of manifestation or intervention, e.g. myocardial infarction, atrial fibrillation or symptomatic arrhythmias, heart failure, positive cardiac family history for CHD), cerebrovascular disease (transient ischaemic attack, prolonged ischaemic neurological deficit, and stroke), renal insufficiency, other risk factors (hypercholesterolaemia, arterial hypertension, smoking, microalbuminuria). If applicable, general information on educational measures for patients about DM (on lifestyle changes and diet), coagulation (vitamin K antagonists), arterial hypertension or other, were noted.

Current therapy was recorded for beta-blockers, antiplatelets, nitrates, ACE inhibitors, calcium antagonists, oral antidiabetic drugs, and insulin. Lipid-lowering drugs were recorded, with particular focus on statins (simvastatin, lovastatin, fluvastatin, pravastatin, atorvastatin) with the respective dosages (10, 20, 40, 80 mg/d). Further, the cholesterol absorption inhibitor ezetimibe, fibrates, nicotinic acid derivatives and bile acid sequestrants were recorded.

The results of the current treatment were noted for hyperlipidaemia (laboratory values for total cholesterol, LDL-C, high density lipoprotein cholesterol (HDL-C), and triglycerides), for hypertension (systolic and diastolic blood pressure), and long-term glycaemia status (HbA_1c_). Physicians commented on whether, according to their judgement, LDL-C target levels were attained ("individual targets").

At about 4 months, drug therapy and results were recorded in an analogous manner as at entry. Apart from these data, no further information about efficacy and safety was collected. If an adverse drug reaction occurred, physicians were requested to notify the manufacturer of the drug associated with the event.

### Data management and statistics

Data were stored with the database system Microsoft Access 2003, and analysed with the statistical program SAS release 8.2. (SAS Institute Inc., Cary, NC, USA). For quality assurance, plausibility checks using minimum and maximum values for the individual parameters were applied. Descriptive statistics were calculated and distribution of parameters was presented as means with standard deviation. Data are presented by indication (DM vs. CHD) and by DMP versus non-DMP groups, respectively. Statistical comparisons were performed between patients in the DMP vs. non-DMP groups within the two indications (statistical significance was set at the 0.05 level). For this descriptive analysis, corrections for multiple comparisons were not performed.

## Results

### Physician characteristics

The majority of investigators (n = 6551) were general/family physicians (72.2%) or internists (30.1%). A specialisation in diabetology was reported in 3.9%, and in cardiology in 1.8%. Most physicians (60.8%) worked alone, and 36.4% in various cooperation forms with colleagues (2.8% not reported). Of the physicians, 24.7% saw ≥ 1500 patients/quarter with insurance in sick funds, 36.8% between 1000 and 1500, and 26.2% fewer than 1000.

The large majority of physicians (93.1%) took part in a DMP: 25.7% unspecified, 61.6% in both DMPs for CHD and DM, 10.3% for DM alone, and 2.5% for CHD alone. Practices that did not participate in any DMP accounted for 6.7% (0.2% not reported).

### Patient characteristics and comorbidities at entry

A total of 45,873 patients were documented in the registry. Patients with private insurance (n = 3047), those without information on DMP status (n = 747), patients who participated in both DMP programs concomitantly (n = 8233 patients), and those with no inclusion diagnosis (n = 4630) were not considered for this analysis.

**Figure 1:**
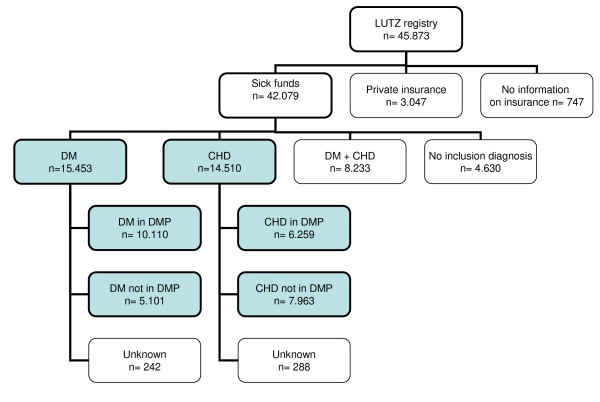
**CHD, coronary heart disease; DM-2, type 2 diabetes mellitus; DMP, disease management program**. Results of patients in shadowed fields are described in detail in the tables of this paper. Note that results of patients who participated in *both DMPs concomitantly *(n = 8233) are not described in the present article.

Figure 1 displays the distribution of patients by indication and by their participation in one of the DMPs. While two thirds of DM patients were registered in respective DMP, less than half of CHD patients were.

Table 1 shows the demographic characteristics of patients at baseline. As expected, concomitant diseases and risk factors were frequent in patients with DM as well as CHD. No important differences were noted with regards to age, gender distribution, and body mass index between DMP and non-DMP patients. However, with regards to concomitant diseases, the DM DMP patients had a higher rate of comorbidities for almost all concomitant diseases. In the CHD indication, for DMP patients there was a substantial higher proportion of patients with post myocardial infarction or coronary artery bypass graft, while the non-cardiac comorbidities did not differ relevantly between DMP and non-DMP patients (Table 1 bottom).

**Table 1:** Demographics, comorbidities and cardiovascular risk factors, by indication and DMP participation, respectively

	DM in DMP		DM not in DMP		
**Parameter**	**Value**	n/N	**Value**	n/N	p-value

	Mean ± SD	10110	Mean ± SD	5101	

Age (years)					

All	62.1 ± 11.4		62.4 ± 11.8		0.1

Male	60.8 ± 11.0		60.5 ± 11.3		<0.0001

Female	63.6 ± 11.7		64.4 ± 11.9		<0.0001

BMI (kg/m^2^)					

All	30.1 ± 5.2		29.6 ± 5.0		<0.0001

Male	29.8 ± 4.8		29.4 ± 4.6		<0.05

Female	30.5 ± 5.6		29.8 ± 5.4		<0.05

**Cardiovascular risk factors**	**%**		**%**		

Hypercholesterolemia	88.3	(8932/10110)	88.7	(4525/5101)	0.51

Hypertension	83.5	(8445/10110)	78.5	(4002/5101)	<0.0001

Current smoker	17.5	(1774/10110)	19.8	(1011/5101)	<0.001

Positive family history for CHD	24.3	(2456/10110)	21.8	(1110/5101)	<0.001

**Comorbidity**	**%**		**%**		

Angina pectoris	7.7	(779/10110)	5.5	(280/15101)	<0.0001

Myocardial infarction	6.4	(652/10110)	3.6	(184/5101)	<0.0001

Coronary artery bypass graft (CABG)	3.7	(370/10110)	1.6	(83/5101)	<0.0001

Cardiac insufficiency	9.4	(955/10110)	9.2	(469/5101)	0.61

Atrial fibrillation cardiac arrhythmia	6.3	(633/10110)	5.4	(275/5101)	<0.05

Renal insufficiency	7.7	(774/10110)	6.9	(352/5101)	0.09

Microalbuminuria	16.1	(1630/10110)	13.0	(662/5101)	<0.0001

Peripheral arterial disease (PAD)	7.9	(796/10110)	6.7	(340/5101)	0.01

Peripheral amputation due to PAD	1.1	(111/10110)	1.1	(54/5101)	0.83

Stroke/transient ischemic ischemia	6.7	(682/10110)	6.4	(327/5101)	0.43

	**CHD in DMP**		**CHD not in DMP**		

**Parameter**	**Value**	n/N	**Value**	n/N	p-value

	Mean ± SD	6259		7963	

Age (years)					

All	62.6 ± 11.5		63.7 ± 11.5		<0.0001

Male	62.0 ± 10.9		62.4 ± 11.2		<0.0001

Female	63.8 ± 12.5		65.8 ± 11.7		<0.0001

BMI (kg/m^2^)					

All	27.5 ± 3.8		27.6 ± 3.9		0.74

Male	27.6 ± 3.6		27.6 ± 3.7		<0.001

Female	27.3 ± 4.3		27.4 ± 4.2		<0.001

**Cardiovascular risk factors**	**%**		**%**		

Hypercholesterolemia	92.0	(5760/6259)	92.4	(7355/7963)	0.46

Hypertension	83.7	(5236/6259)	81.4	(6483/7963)	<0.001

Current smoker	22.3	(1393/6259)	24.9	(1983/7963)	<0.001

Positive family history for CHD	39.2	(2452/6259)	40.0	(3184/7963)	0.33

**Comorbidity**	**%**		**%**		

Angina pectoris	31.3	(1960/6259)	31.2	(2493/7963)	0.86

Myocardial infarction	54.1	(3384/6259)	37.5	(2984/7963)	<0.0001

Coronary artery bypass graft (CABG)	31.9	(1994/6259)	21.8	(1734/7963)	<0.0001

Cardiac insufficiency	14.7	(917/6259)	13.2	(1051/7963)	<0.05

Atrial fibrillation cardiac arrhythmia	11.1	(697/6259)	11.8	(943/7963)	0.19

Renal insufficiency	5.0	(310/6259)	4.7	(378/7963)	0.57

Microalbuminuria	2.6	(164/6259)	2.3	(186/7963)	0.28

Peripheral arterial disease (PAD)	7.7	(483/6259)	8.0	(640/7963)	0.48

Peripheral amputation due to PAD	0.2	(15/6259)	0.3	(25/7963)	0.41

Stroke/transient ischemic ischemia	5.1	(318/6259)	6.6	(527/7963)	<0.001

DMP, Disease Management Program; SD, standard deviation; DM, diabetes mellitus; CHD, coronary heart disease. n, number of documented patients; N, all patients in the group. Difference to 100%: value not reported. * p-values refer to difference between patient in DMP and not in DMP (routine care) in the respective indication.

### Educational measures and drug therapy

In the DM indication, DMP patients compared to non-DMP patients received more frequently educational measures for lifestyle and diet (75.6% vs. 40.7%) or hypertension (12.0% vs. 7.4%). Regarding CHD, a similar finding was noted for educational measures on hypertension (27.0% vs. 14.0%).

Table 2 summarises the disease-specific drug therapy at inclusion at 4 months. With declining frequency, statins, beta-blockers, and ACE inhibitors were the most frequently named classes. Regarding DM, in DMP patients all cardiac and antidiabetic medications were more frequently reported in DMP compared to non-DMP patients, but no major differences were noted for lipid lowering medications. Similarly, regarding CHD, all drug classes were at least numerically more frequent in DMP patients (with the exception of fibrates), but the differences for lipid lowering medications were generally small (for example statins in CHD: 81.4% vs. 79.4%). The most frequently prescribed agent was simvastatin. The 20 mg and 40 mg daily dose were preferably used for all statins. Notably, only about a quarter of DM patients were prescribed antiplatelets, in contract to three quarters of CHD patients.

**Table 2:** Diagnosis specific medication at inclusion and after 4 months

Parameter	Type-2 Diabetes Mellitus				
	**DM in DMP** **n = 10110**		**DM not in DMP** **n = 5101**		

	**Inclusion**	**After 4 months**	**Inclusion**	**After 4 months**	p-value*

**Drug class/agent**	%	%	%	%	

					

Beta blocker	**42.8**	41.2	**40.1**	39.8	<0.01

Antiplatelet	**27.0**	26.7	**23.8**	23.3	<0.0001

Nitrate	**5.5**	5.5	**4.0**	4.0	<0.0001

ACE inhibitor	**61.3**	58.5	**55.5**	52.8	<0.0001

Calcium antagonist	**23.8**	23.0	**21.3**	19.9	<0.001

Oral antidiabetic drug	**67.8**	62.2	**63.9**	58.9	<0.0001

Insulin	**31.9**	29.9	**22.8**	20.8	<0.0001

Statin monotherapy	**77.5**	57.7	**76.8**	57.0	0.3

Simvastatin	**53.5**	38.3	**52.5**	36.7	0.27

Lovastatin	**2.2**	1.3	**2.8**	1.6	<0.05

Pravastatin	**9.2**	5.5	**8.7**	5.7	0.3

Fluvastatin	**5.2**	3.8	**4.8**	3.7	0.38

Atorvastatin	**3.3**	1.8	**3.3**	1.9	0.92

Ezemtimibe+ statin combination	**12.0**	31.0	**12.2**	31.3	0.79

Ezetimibe monotherapy	**1.6**	2.4	**1.8**	2.6	0.39

Fibrates	**3.0**	2.2	**2.8**	2.1	0.48

Nicotinic acid derivates	**0.5**	0.5	**0.3**	0.5	0.12

Anionic-exchange resins	**0.0**	0.0	**0.1**	0.1	0.19

**Parameter**	**Coronary Heart Disease**				

	**CHD in DMP** **n = 6259**		**CHD not in DMP** **n = 7963**		

	**Inclusion**	**After 4 months**	**Inclusion**	**After 4 months**	p-value*

**Drug class/agent**	%	%	%	%	

					

Beta blocker	**81.7**	76.5	**71.6**	67.6	<0.0001

Antiplatelet	**63.0**	58.5	**53.6**	49.5	<0.0001

Nitrate	**24.2**	22.0	**18.6**	16.9	<0.0001

ACE inhibitor	**68.1**	64.4	**64.1**	60.0	<0.0001

Calcium antagonist	**20.9**	19.6	**20.6**	19.3	0.67

Oral antidiabetic drug	**3.2**	4.6	**2.4**	3.5	<0.01

Insulin	**1.1**	1.2	**0.9**	0.9	0.4

Statin monotherapy	**81.4**	57.9	**79.4**	53.3	<0.01

Simvastatin	**52.4**	35.7	**48.6**	31.3	<0.0001

Lovastatin	**2.3**	1.3	**2.2**	1.5	0.75

Pravastatin	**9.5**	5.7	**10.4**	5.8	0.09

Fluvastatin	**6.5**	4.4	**6.4**	4.0	0.86

Atorvastatin	**6.5**	3.9	**7.0**	3.8	0.28

Ezemtimibe+ statin combination	**17.2**	37.4	**16.8**	41.2	0.6

Ezetimibe monotherapy	**3.1**	4.0	**3.0**	3.5	0.84

Fibrates	**1.1**	0.8	**1.6**	1.0	<0.05

Nicotinic acid derivates	**0.6**	0.6	**0.6**	0.7	0.99

Anionic-exchange resins	**0.1**	0.1	**0.1**	0.1	0.81

Values are mean ± SD or percentages, respectively. ACE inhibitor, angiotensin converting enzyme inhibitor.

*p-values refer to difference between patient in DMP and not in DMP (routine care) in the respective indication **at inclusion**.

At 4 months, the rate of patients with a statin/ezetimibe combination therapy increased, whereas treatment rates with respect to almost all other drug classes decreased slightly.

### Target level attainment

#### Lipids

At entry, mean LDL-C values were significantly lower in DMP patients in DMPs compared to patients in routine care (DM: 130 mg/dl vs. 134 mg/dl; CHD: 122 mg/dl vs. 132 mg/dl). At entry, LDL-C target level attainment (< 100 mg/dl) was generally low, however, higher rates in DMP patients were noted compared to non-DMP patients with a much larger difference in CHD patients (DM: 23.9% vs. 21.3%; CHD: 30.6% vs. 23.8%; Table 3).

**Table 3:** Lipids, HbA1c and blood pressure: values and target level attainment at inclusion and after 4 months

Parameter	Type-2 Diabetes Mellitus				
	**DM in DMP** **n = 10110**		**DM not in DMP** **n = 5101**		p-value*

Lipids	**Inclusion**	**After 4 months**	**Inclusion**	**After 4 months**	

LDL-C; mg/dl (mean ± SD)	129.5 ± 38.9	111.9 ± 30.4	133.5 ± 40.4	113.5 ± 31.1	<0.0001

LDL-C < 100 mg/dl	23.9%	38.3%	21.3%	36.9%	<0.01

LDL-C target level attained**	46.5%	59.6%	35.0%	56.5%	<0.0001

LDL-C target level not attained**	48.9%	30.9%	59.5%	32.4%	<0.001

					

Total cholesterol, mg/dl (mean ± SD)	218.1 ± 50.5	195.5 ± 38.7	223.8 ± 52.2	198.0 ± 39.5	<0.0001

HDL-C, mg/dl (mean ± SD)	51.2 ± 13.3	52.4 ± 12.8	51.0 ± 13.2	52.3 ± 12.6	0.49

Triglycerides, mg/dl (mean ± SD)	199.7 ± 96.7	179.5 ± 82.4	201.6 ± 95.9	181.7 ± 84.5	0.11

					

Blood glucose					

HbA_1c_, % (mean ± SD)	7.1 ± 1.1	6.9 ± 1.0	7.1 ± 1.2	6.8 ± 1.0	<0.001

					

Blood pressure, mmHg					

Systolic (mean ± SD)	138.1 ± 14.9	134.7 ± 13.4	138.8 ± 15.0	134.8 ± 13.4	<0.05

Diastolic (mean ± SD)	81.3 ± 8.5	79.9 ± 7.8	81.8 ± 8.7	80.3 ± 7.8	<0.001

**Parameter**	**Coronary Heart Disease**				

	**CHD in DMP** **n = 6259**		**CHD not in DMP** **n = 7963**		p-value*

Lipids	**Inclusion**	**After 4 months**	**Inclusion**	**After 4 months**	

LDL-C; mg/dl (mean ± SD)	122.1 ± 38.0	104.6 ± 27.7	131.6 ± 41.7	109.7 ± 30.9	<0.0001

LDL-C < 100 mg/dl	30.6%	49.8%	23.8%	43.3%	<0.0001

LDL-C target level attained**	42.4%	65.7%	34.5%	60.6%	<0.0001

LDL-C target level not attained**	53.8%	25.7%	61.8%	30.2%	<0.0001

					

Total cholesterol, mg/dl (mean ± SD)	206.5 ± 48.7	184.8 ± 35.8	219.9 ± 54.0	192.7 ± 39.6	<0.0001

HDL-C, mg/dl (mean ± SD)	51.4 ± 12.8	52.3 ± 12.3	52.4 ± 13.7	53.6 ± 13.1	<0.01

Triglycerides, mg/dl (mean ± SD)	170.9 ± 85.1	156.7 ± 72.1	178.1 ± 87.8	160.0 ± 74.1	<0.0001

					

Blood glucose					

HbA_1c_, % (mean ± SD)	6.2 ± 0.9	6.2 ± 0.8	6.1 ± 0.9	6.1 ± 0.8	0.09

					

Blood pressure, mmHg					

Systolic (mean ± SD)	132.6 ± 14.6	130.3 ± 12.9	135.2 ± 15.1	131.8 ± 12.7	<0.0001

Diastolic (mean ± SD)	79.6 ± 8.3	78.7 ± 7.7	80.9 ± 8.6	79.6 ± 7.6	<0.0001

* p-values refer to difference between DMP and non-DMP patients in the respective indication **at inclusion**

** according to physician assessment (not according to guidelines)

After 4 months, rates had improved substantially in DMP patients and non-DMP patients (DM: 38.3% vs 36.9%; CHD: 49.8% vs. 43.3%). Notably, investigator's ratings of *individual *LDL-C target level attainment rates were substantially higher in all groups.

Mean total cholesterol and triglyceride values decreased substantially, while HDL-C values increased slightly both in DMP and non-DMP patients (in DM and CHD).

#### Blood pressure and HbA_1c_

Compared to baseline values, mean systolic/diastolic blood pressure were slightly lower after 4 months, with no substantial differences between DMP and non-DMP patients (Table 3 bottom). Likewise, in diabetic patients mean HbA_1c _decreased slightly from a baseline level of 7.1% to 6.9%.

## Discussion

### Characteristics and comorbidities

The present registry documents a large current sample of primary care patients with type 2 DM or CHD, managed in the context of DMPs or in routine care (non-DMP). While patients in the respective DMPs did not differ remarkably in terms of demographic characteristics (age, gender, BMI), they did with regards to comorbidities (which were generally more frequent in DMP patients). The present data do not confirm the concern that in the DMPs the relatively young and healthy diabetics would be enrolled rather than the targeted high-risk population [12]. A substantial difference between DMP and non-DMP patients was for the former group the higher participation rate in patient education programs, which can be attributed, among other factors, to the explicit recommendations stated in the DMP guidelines. It was in the same order for DM (but lower for CHD) when compared to a nationwide cross-sectional survey of primary care in Germany in 2003, which reported a rate of 65.0% for diet counselling, dietary education, or physical activity education programs for DM patients versus 52.5% for CHD patients [13].

### Treatment and target level attainment

In terms of treatment, DMP patients (DM and CHD) had higher drug prescription rates than non-DMP patients, which may reflect higher treatment intensity, but may also correspond to the higher rates of comorbidities. For CHD patients in DMPs a similar finding has been reported from the ELSID study [14]. It has in fact been shown that in DM patients treatment intensity is generally increased after complications have occurred, i.e. at a later stage in the disease process [15]. While treatment rates with antiplatelet drugs were higher in DMP patients, they were not satisfactory in any subgroup, as patients with CHD and those with DM (as a coronary equivalent) are at high risk of a recurrent or first cardiovascular event and should receive such drugs [16,17].

A focus of this registry was on the treatment with lipid-lowering drugs. The NCEP ATP III guidelines re-emphasised the importance of lowering elevated levels of LDL-C as the most efficacious treatment to reduce the incidence of CHD mortality and morbidity [11,18]. For every 30 mg/dl change in LDL-C the relative risk for the incidence of coronary artery disease (CAD) changes by 30% [11]. Consequently, for patients with a high cardiovascular risk (e.g. with manifest CHD or DM) a LDL-C target of < 100 mg/dl (2.6 mmol/l) should be reached, and in patients with very high CHD risk, optionally < 70 mg/dl [11]. The present registry shows that a high percentage of patients, irrespective of inclusion in DMPs, do not attain these lipid targets. This is in line with reports from earlier registries in Germany, for example the DUTY registry (2002) in 59,035 patients with DM (LDL-C target attainment rate < 100 mg/dl 16.6% at the end of observation) [19], the 4E registry (2001/2002) in the subgroup of 12,816 patients with DM in primary or secondary CHD prevention (LDL-C target attainment rates < 100 mg/dl 16% in men, 12% in women) [20], and the 2L cardio registry in high-risk or very-high-risk patients (37.1% < 100 mg/) [21]. No effectiveness data on the lipid management are available in the context of DMPs in Germany to date, but reports from the US in the managed care environment show that the situation is similarly suboptimal [22-24]. It must be noted that in the German DMPs for CHD the target value for LDL-C is set at 100 mg/dl, while in the DMPs for DM, lipid-lowering therapy is recommended, but no target value provided [4]. This may provide one explanation, why in this registry the LDL-C target attainment in patients in the CHD DMP was slightly higher (at entry and follow-up) than in those in the DM DMP. Another explanation is that patients with manifest CHD are more likely to be stringently treated than those with a coronary risk equivalent "only".

Patients irrespective of inclusion in the DMPs improved with regard to their lipid status during the follow-up in our registry. The effect was substantial, as through intensification of therapy an additional LDL-C decrease of 21 mg/dl was achieved. Of note, the rates of combination therapy with statins plus ezetimibe increased substantially. NCEP ATP III describe various options to achieve stringent lipid targets, among them high doses of statins, but also various combination approaches of statins with ezetimibe, fibrates, bile acid sequestrants, or nicotinic acid, under consideration to safety of the regimen for the individual patient as well as to efficacy of treatment [11]. It has repeatedly been reported that physicians are reluctant to increase statin doses, for example in the managed care environment where titration was noted in only 3% [25], or in other settings [26,27]. Our registry data were in line with these findings, as mean doses of the various statins were in the lower range of the labelled range, were only slightly increased during the follow-up, and the full 80 mg doses were rarely prescribed. The reluctance to prescribe statins in high doses may be due to the fear of increased side effects [28].

Mean BP and HbA_1c _during the 4-month follow-up were only slightly modified, which hints at the need of further treatment intensification. The HYDRA study in 2003 has shown that only a marginal proportion (1.3%) of all diabetic patients achieves a combined target of LDL < 100 mg/dl, HbA1c < 7% and blood pressure < 135/85 mmHg [29].

### Methodological considerations

A number of methodological considerations deserve attention. A more optimal design for addressing our research questions would have been a (cluster-)randomised approach (DMP versus routine care) [14]. Further, the sample selection was mainly guided by hypercholesterolemia as the qualifying diagnosis and statins as treatment, which is a selection process. Comparisons between groups can only be made with caution (potential confounding by comorbidity), which is a methodological problem applying to all analysis of the results of DMPs [30]. The distribution of guidelines can to some extent be regarded to be a form of intervention, since physicians were informed about the LDL-C targets, which may well have contributed to the substantial improvement in target level achievement rates, irrespective of the participation in a DMP. The observation period of 4 months is rather short in view of the long-term management that is indicated for these patients, but it shows that within a short time frame, substantial improvement in lipid management can be achieved. Even so, in addition to the surrogate laboratory measures, disease-specific endpoints, in particular cardiovascular outcomes, would be of great interest [31]. Preliminary data from the first randomised controlled study in 2300 patients enrolled in diabetes DMPs in 85 primary care offices suggest that mortality in these patients compared to routine care patients matched for demographic characteristics and severity of disease might be reduced [30].

## Conclusion

In summary, the present registry shows that patients in DMPs do not relevantly differ from non-DMP patients with regards to demographic characteristics, but have a higher level of comorbidity. DMP patients receive more intensive drug and non-drug treatment (educational measures), and have generally more favourable lipid levels and slightly higher target LDL-C attainment rates (which must be seen in the context of higher baseline LDL-C values for non-DMP patients). However, for BP and HbA_1c_, the participation in DMP has no impact. The distribution of the NCEP ATP III guidelines as a reminder on lipid target levels generally appears to be suitable for improving LDL-C values.

Overall, at the end of the follow-up period, mean LDL-C target attainment rates in DMP and non-DMP patients were among the highest reported to date in primary care, which is an encouraging finding.

## Competing interests

KB and CJ are employees of MSD SHARP & DOHME GMBH, Germany, and BK is an employee of Essex Pharma GmbH, Germany. Both companies are manufacturers of ezetimibe and simvastatin, which are used, among others, in the indication hypercholesterolaemia. The other authors declare that they have no competing interests.

## Authors' contributions

KB, CJ, and BK designed the study and interpreted the results. DP and WK contributed to the analyses and the interpretation of results. KB and DP wrote the paper. All authors read and approved the final manuscript.

## Pre-publication history

The pre-publication history for this paper can be accessed here:

http://www.biomedcentral.com/1471-2458/9/280/prepub

## References

[B1] Faxon DP Schwamm LH Pasternak RC Peterson ED McNeil BJ Bufalino V Yancy CW Brass LM Baker DW Bonow RO Improving quality of care through disease management: principles and recommendations from the American Heart Association's Expert Panel on Disease Management Circulation 2004 9 21 2651 2654 10.1161/01.CIR.0000128373.90851.7B 15173048

[B2] Epstein RS Sherwood LM From outcomes research to disease management: a guide for the perplexed Ann Intern Med 1996 9 9 832 837 8610953 10.7326/0003-4819-124-9-199605010-00008

[B3] Krumholz HM Currie PM Riegel B Phillips CO Peterson ED Smith R Yancy CW Faxon DP A taxonomy for disease management: a scientific statement from the American Heart Association Disease Management Taxonomy Writing Group Circulation 2006 9 13 1432 1445 10.1161/CIRCULATIONAHA.106.177322 16952985

[B4] Busse R Disease management programs in Germany's statutory health insurance system Health Aff (Millwood) 2004 9 3 56 67 10.1377/hlthaff.23.3.56 15160803

[B5] Beyer M Gensichen J Szecsenyi J Wensing M Gerlach FM [Effectiveness of German disease management programs ‐ problems of clinical evaluation research in the light of a study protocol] Z Arztl Fortbild Qualitatssich 2006 9 5 355 363 16955621

[B6] Bundesversicherungsamt (BVA) [Accreditaton of Disease Management Programs (DMP) by the Bundesversicherungsamt (BVA)] http://www.bundesversicherungsamt.de/**Accessed on 23 July 2009**

[B7] Bundesversicherungsamt (BVA) [Summary of evaluation results for DMPs diabetes mellitus type II, 2003‐2006]. Original in German http://www.bundesversicherungsamt.de/cln_100/nn_1046648/DE/DMP/Downloads/Evaluationsergebnisse__Diabetes__03-06,templateId=raw,property=publicationFile.pdf/Evaluationsergebnisse_Diabetes_03-06.pdf **Accessed on 23 July 2009**

[B8] Greiner W [Health economic evaluation of disease management programs] Bundesgesundheitsblatt Gesundheitsforschung Gesundheitsschutz 2006 9 1 34 39 10.1007/s00103-005-1193-4 16320012

[B9] Bundesministerium für Gesundheit Die neue Gesundheitsversicherung http://www.die-gesundheitsreform.de **Accessed on 23 July 2009**

[B10] Dietrich E Jopp R Schreier U Gilge R Bartmann P Berthold H Drug Expenditures Resulting from the Implementation of Clinical Practice Guidelines in Germany [article in German] Gesundh ökon Qual manag 2005 9 35 43 10.1055/s-2004-813943

[B11] Grundy SM Cleeman JI Merz CN Brewer HB Jr Clark LT Hunninghake DB Pasternak RC Smith SC Jr Stone NJ Implications of recent clinical trials for the National Cholesterol Education Program Adult Treatment Panel III guidelines Circulation 2004 9 2 227 239 10.1161/01.CIR.0000133317.49796.0E 15249516

[B12] Bullmann C Straub C [Disease management programs between aspiration and reality. Actually, everything was meant to become much better] Z Arztl Fortbild Qualitatssich 2006 9 1 32 35 discussion 36 16524227

[B13] Pittrow D Pieper L Klotsche J Wittchen H eds DETECT. Ergebnisse einer klinisch-epidemiologischen Querschnitts- und Verlaufsstudie mit 50.000 Patienten in 3.000 Hausarztpraxen 2007 Elsevier, München

[B14] Joos S Rosemann T Heiderhoff M Wensing M Ludt S Gensichen J Kaufmann-Kolle P Szecsenyi J ELSID-Diabetes study-evaluation of a large scale implementation of disease management programmes for patients with type 2 diabetes. Rationale, design and conduct ‐ a study protocol [ISRCTN08471887] BMC Public Health 2005 9 99 10.1186/1471-2458-5-99 16202151 PMC1260025

[B15] Pittrow D Stalla GK Zeiher AM Silber S Marz W Pieper L Klotsche J Glaesmer H Ruf G Schneider HJ [Prevalence, drug treatment and metabolic control of diabetes mellitus in primary care] Med Klin (Munich) 2006 9 8 635 644 10.1007/s00063-006-1093-x 16896570

[B16] Ryden L Standl E Bartnik M Berghe G Van den Betteridge J de Boer MJ Cosentino F Jonsson B Laakso M Malmberg K Guidelines on diabetes, pre-diabetes, and cardiovascular diseases: executive summary: The Task Force on Diabetes and Cardiovascular Diseases of the European Society of Cardiology (ESC) and of the European Association for the Study of Diabetes (EASD) Eur Heart J 2007 9 1 88 136 10.1093/eurheartj/ehm124 17220161

[B17] Smith SC Jr Allen J Blair SN Bonow RO Brass LM Fonarow GC Grundy SM Hiratzka L Jones D Krumholz HM AHA/ACC Guidelines for Secondary Prevention for Patients With Coronary and Other Atherosclerotic Vascular Disease: 2006 Update: Endorsed by the National Heart, Lung, and Blood Institute Circulation 2006 9 19 2363 2372 10.1161/CIRCULATIONAHA.106.174516 16702489

[B18] Abildstrom SZ Rask-Madsen C Ottesen MM Andersen PK Rosthoj S Torp-Pedersen C Kober L Impact of age and sex on sudden cardiovascular death following myocardial infarction Heart 2002 9 6 573 578 10.1136/heart.88.6.573 12433881 PMC1767447

[B19] Krone W Böhm M Wöhrmann A Bestehorn K Erhebung und Verbesserung der Behandlungssituation von Patienten mit Diabetes mellitus. Das DUTY-Register Bundesgesundheitsblatt ‐ Gesundheitsforschung ‐ Gesundheitsschutz 2004 9 6 540 546 15221103 10.1007/s00103-004-0843-2

[B20] Assmann G Schulte H Cullen P Neiss A Bestehorn K Treatment of hyperlipidemia in primary practise in Germany: sub-group analyses from the 4E-registry with particular emphasis on men and women with diabetes mellitus Exp Clin Endocrinol Diabetes 2007 9 2 85 91 10.1055/s-2007-955094 17318766

[B21] Bestehorn K Gitt AK Jünger C Senges J Guideline-oriented therapy in outpatients with hypercholesterolemia: 2L registry. Stiftung Institut für Herzinfarktforschung. Abschlußbericht vom 1.8.2007 (data on file)

[B22] Meyer JW Schultz JS O'Donnell JC Patel PA Sasane RM Patterns and effectiveness of lipid-lowering therapies in a managed care environment Value Health 2005 9 5 601 612 10.1111/j.1524-4733.2005.00052.x 16176498

[B23] Nag SS Daniel GW Bullano MF Kamal-Bahl S Sajjan SG Hu H Alexander C LDL-C goal attainment among patients newly diagnosed with coronary heart disease or diabetes in a commercial HMO J Manag Care Pharm 2007 9 8 652 663 17970603 10.18553/jmcp.2007.13.8.652 PMC10437400

[B24] Stacy TA Egger A Results of retrospective chart review to determine improvement in lipid goal attainment in patients treated by high-volume prescribers of lipid-modifying drugs J Manag Care Pharm 2006 9 9 745 751 17249907 10.18553/jmcp.2006.12.9.745 PMC10437990

[B25] Valuck RJ Williams SA MacArthur M Saseen JJ Nair KV McCollum M Ensor JE A retrospective cohort study of correlates of response to pharmacologic therapy for hyperlipidemia in members of a managed care organization Clin Ther 2003 9 11 2936 2957 10.1016/S0149-2918(03)80346-6 14693317

[B26] McKenney JM Optimizing LDL-C lowering with statins Am J Ther 2004 9 1 54 59 10.1097/00045391-200401000-00011 14704596

[B27] Ose L Skjeldestad FE Bakken IJ Levorsen A Alemao EA Yin DD Borgstrom F Jonsson L Lipid management and cholesterol goal attainment in Norway Am J Cardiovasc Drugs 2006 9 2 121 128 10.2165/00129784-200606020-00006 16555865

[B28] Law MR Wald NJ Rudnicka AR Quantifying effect of statins on low density lipoprotein cholesterol, ischaemic heart disease, and stroke: systematic review and meta-analysis Bmj 2003 9 7404 1423 10.1136/bmj.326.7404.1423 12829554 PMC162260

[B29] Pittrow D Medikamente gegen Bluthochdruck und Diabetes mellitus. Pharmakoepidemiologische Aspekte bei der Verordnung von Antihypertensiva und Antidiabetika in der primärärztlichen Versorgung in Deutschland. 1. Auflage 2005 Aachen: Shaker

[B30] AOK Bundesverband Evaluation of a Large Scale Implementation of Disease Management Programmes (ELSID). Bonn, 12. August 2008 http://www.presseportal.de/pm/8697/1245089/aok_bundesverband/**Accessed on 23 July 2009**

[B31] Campbell SM Braspenning J Hutchinson A Marshall MN Improving the quality of health care: Research methods used in developing and applying quality indicators in primary care Brit Med J 2003 9 7393 816 819 10.1136/bmj.326.7393.816 12689983 PMC1125721

